# Allergenic and cytotoxic potential of (meth)acrylate monomers in dental materials: a narrative review

**DOI:** 10.3389/fdmed.2026.1846292

**Published:** 2026-06-24

**Authors:** Lukas Smidtas, Jaylan Johnson, Rafael Rocha Pacheco, Catherine Jauregui

**Affiliations:** 1 Dental College of Georgia at Augusta University, Augusta, GA, United States; 2 Department of Restorative Sciences, Dental College of Georgia at Augusta University, Augusta, GA, United States; 3 Department of Oral Biology, Dental College of Georgia at Augusta University, Augusta, GA, United States

**Keywords:** dental materials, dermatitis, allergic contact, occupational, hypersensitivity, delayed, methacrylate, occupational exposure, polymerization

## Abstract

This narrative review synthesizes current evidence on the allergenic and cytotoxic potential of (meth)acrylate monomers. Residual monomers act as haptens, triggering type IV (delayed-type) hypersensitivity reactions in both patients and dental professionals. Exposure occurs through dermal contact, oral mucosa, ocular tissues, and inhalation of nanoparticles generated during grinding and polishing. Standard dental gloves provide limited chemical protection. Cross-sensitization among structurally related monomers is common, and previous sensitization through non-dental sources such as nail products or industrial adhesives may predispose individuals to reactions from dental materials. The rapid adoption of additive manufacturing introduces additional concerns, as 3D-printed resins demonstrate significant variability in polymerization quality depending on post-processing protocols, and standardized guidelines remain lacking. Protective strategies should follow a hierarchy of controls approach, prioritizing safer monomer formulations, engineering controls, and no-touch techniques over personal protective equipment alone. Enhanced awareness, comprehensive allergy histories, and the development of low-allergenicity alternatives are essential to mitigate risks as (meth)acrylate use continues to expand.

## Clinical implications

1. Change gloves every 15–20 min when handling uncured resins; no glove type provides reliable chemical protection.
2. Ask patients about prior reactions to nail products, adhesives, or medical devices.
3. Ensure adequate ventilation when operating 3D printers and during post-processing.
4. Recognize early sensitization signs promptly; once established, (meth)acrylate sensitivity is typically permanent.
5. Prioritize engineering controls (ventilation, unit-dose systems) and no-touch techniques over PPE alone.

## Introduction

Synthetic polymers, particularly (meth)acrylate-based materials, have been used in dentistry since the early 20th century, forming the basis of many restorative, prosthetic, and adhesive systems (1). Their continued use reflects favorable mechanical and esthetic properties, ease of manipulation, and overall cost-effectiveness (1–5). However, the role of (meth)acrylates has expanded dramatically in the 21st century, driven by advances in adhesive dentistry, growing patient demand for esthetic restorations, and the emergence of digital workflows. Today, (meth)acrylates are incorporated into restorative systems (composites, sealants, bonding agents), prosthetic devices (denture bases, temporary restorations, maxillofacial prostheses), and orthodontic appliances (1, 2, 6, 7). Digital manufacturing has further accelerated their use through both subtractive manufacturing (CAD-CAM milling of pre-polymerized resin blocks) and additive manufacturing (3D printing with photopolymer resins) (7). As a result, both patients and dental professionals now experience more frequent and prolonged exposure to methacrylate-based materials due to the widespread use of resin-based dental products (8).

Common monomers such as 2-hydroxyethyl methacrylate (HEMA), triethylene glycol dimethacrylate (TEGDMA), bisphenol-A glycidyl dimethacrylate (Bis-GMA), and urethane dimethacrylate (UDMA) are incorporated into formulations to tailor properties including viscosity, hydrophilicity, and polymerization kinetics (4, 9). International standards, such as ISO 20795-1 for denture base polymers and ISO 4049 for resin-based filling materials, establish minimum requirements for mechanical strength, residual monomer content, and biocompatibility, providing a framework for assessing clinical suitability (1, 10, 11) (Supplementary Table S1).

Biocompatibility, defined as the ability of a material to perform with an appropriate host response in a specific application, is a key consideration for any dental polymer (2, 12–14). However, a growing body of evidence has documented adverse reactions associated with (meth)acrylate exposure (9). As early as 2004, a national survey in the United Kingdom found that resin composites accounted for over 12% of reported patient reactions to dental materials, while acrylic resins were the leading cause of sensitization among dental technicians (15). In the two decades since, hypersensitivity reactions, particularly allergic contact dermatitis, have been increasingly reported in both patient and occupational settings (2, 6, 16–18). Reflecting this growing clinical significance, acrylates were designated “Allergen of the Year” by the American Contact Dermatitis Society in 2012 (19).

These concerns have prompted further questions about the safety of (meth)acrylates, particularly when materials are used in uncured or partially cured form, in close contact with oral or dermal tissues, or over extended periods (20, 21). Although the biological effects of these monomers have been discussed in isolated prosthodontic, dermatologic, and restorative contexts, the rapid expansion of resin-based digital dentistry and increasing occupational exposure have renewed concerns regarding their cytotoxic and allergenic properties. The existing literature remains segmented across multiple disciplines, limiting access to a clear, singular, consolidated reference for dental professionals. This narrative review critically examines current research on the allergenic potential of (meth)acrylate-based dental materials, summarizing polymerization processes, biological mechanisms of sensitization, clinical manifestations, and routes of exposure, while outlining practical preventive strategies to minimize risk among patients and dental professionals.

## Search strategy

A literature search was conducted in PubMed, ScienceDirect, Wiley Online Library, and the National Institutes of Health online library using combinations of the following terms: “methacrylate,” “acrylate,” “HEMA,” “TEGDMA,” “Bis-GMA,” “UDMA,” “dental materials,” “resin composite,” “denture base,” “allergy,” “hypersensitivity,” “contact dermatitis,” “cytotoxicity,” and “biocompatibility.” Google Scholar was used as a supplementary search tool. No date-restrictions were applied.

Inclusion criteria encompassed peer-reviewed articles published in English, including systematic reviews, meta-analyses, observational studies, narrative reviews, case series, and case reports addressing the biochemical mechanisms, clinical manifestations, or dental relevance of (meth)acrylates. Unpublished studies, conference abstracts and oral or poster presentations were excluded.

Title and abstract screening were performed independently by two authors, with disagreements resolved by consensus. Full-text articles were subsequently reviewed against the inclusion criteria before final selection. Reference lists of included articles were manually screened to identify additional relevant publications. The search extended beyond prosthodontics to include operative dentistry, orthodontics, dental materials science, immunology, and cell biology.

The reliability of included studies was assessed based on study design, with systematic reviews and meta-analyses considered the highest level of evidence, followed by observational studies, narrative reviews, and case-based reports. Although no strict date constraints were initially applied, greater emphasis was placed on papers published within the past 10–15 years to reflect the current understanding of occupational and biological risks, while retaining older foundational studies where they represented primary source material for established mechanistic concepts. Following screening and application of inclusion criteria, 111 references were retained for this review. An additional 6 references were included for regulatory standards, nomenclature, and classification frameworks, for a total of 117 references.

## Polymerization and residual monomer release

In clinical dentistry, polymerization can be initiated through chemical activation (self-curing), light curing (photoactivation), or by heat curing, which is commonly used for laboratory-fabricated prosthetics (1, 20, 22–27). Polymerization proceeds through initiation, propagation, and termination (3, 21, 28); however, the resulting dense polymer network restricts monomer mobility, preventing full conversion (1, 20, 22–24). Consequently, unreacted monomers remain trapped within the resin structure, characterized by degree of conversion (DC) and degree of polymerization (DP), both of which influence the amount of residual monomer available to interact with oral or dermal tissues (1, 4, 21, 29, 30).

### Factors affecting monomer release

No dental polymer currently available achieves complete polymerization (2, 22, 23, 29–31). Laboratory studies report degrees of conversion values ranging from 35% to 95%, depending on the calculation method and experimental conditions (28, 29, 32–35). However, under clinical conditions, DC values for resin composites rarely exceed 75% due to factors such as limited access, suboptimal light tip positioning, operator variability, and restoration depth (21, 23). Other influencing factors include monomer composition, curing method, light intensity, exposure duration, oxygen availability, post-curing protocols, the presence of fillers or inhibitors, and storage conditions (21, 22, 24, 36, 37). Reduced conversion has been consistently associated with increased monomer release and elevated potential for biological exposure (21).

A clinically relevant example is the oxygen-inhibition layer (OIL), a surface region where oxygen interferes with free-radical polymerization, leaving a superficial layer rich in unreacted monomers (37, 38). While the OIL can enhance bonding between resin increments, it also increases the risk of monomer leaching, emphasizing the importance of proper finishing, polishing, and surface treatments such as glycerin gel application during curing (37, 38).

Residual monomers may be released immediately after curing or gradually through degradation and elution (1, 22, 29). Cytotoxicity is highest within the first 24 h following polymerization and decreases over time (4, 20, 22, 28). Factors influencing release within the oral cavity include hydrolytic degradation, enzymatic breakdown by salivary and bacterial esterases, physical wear, temperature fluctuations, and dietary acids (29, 30). The degree of elution is further shaped by molecular weight, solubility, and polymer network density (9). Smaller, more soluble molecules such as HEMA and TEGDMA elute readily, while bulkier monomers like Bis-GMA and UDMA are less prone to leaching due to steric constraints (9, 39) (Table 1). However, clinically relevant toxic exposure thresholds remain difficult to define, because monomer release varies substantially according to material composition, degree of conversion, route of exposure, environmental conditions, and patient-specific biologic susceptibility (9, 29, 30).

**Table 1:** Molecular characteristics of representative dental (meth)acrylate monomers influencing elution behavior.

Monomer	Chemical Name	CAS No.	Molecular Formula	Structure	Molecular Weight (g/mol)	Relative Elution
HEMA	2-Hydroxyethyl methacrylate	868-77-9	C_6_H_10_O_3_	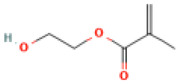	130.14	High
TEGDMA	Triethylene glycol dimethacrylate	109-16-0	C_14_H_22_O_6_	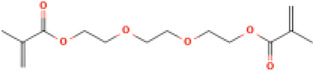	286.32	Moderate-High
UDMA	Urethane dimethacrylate	72869-86-4	C_23_H_38_N_2_O_8_	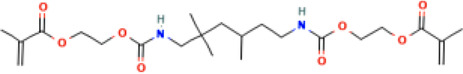	470.56	Moderate
Bis-GMA	Bisphenol A glycidyl methacrylate	1565-94-2	C_29_H_36_O_8_	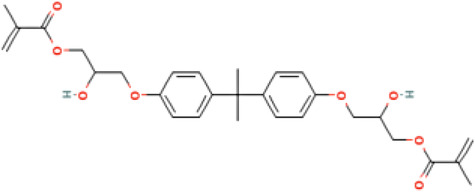	512.59	Low

Structural and molecular data obtained from PubChem (National Institutes of Health). Relative elution potential reflects the inverse relationship between molecular weight and leaching behavior; smaller, more hydrophilic monomers (HEMA) elute more readily than larger, more hydrophobic monomers (Bis-GMA).

## Biological mechanisms of (meth)acrylate sensitization and toxicity

### Immune mechanisms (type IV hypersensitivity)

Residual (meth)acrylate monomers can enter the body through multiple routes, including dermal contact, inhalation, absorption through the oral mucosa, and ocular exposure (2, 16, 17, 20, 40, 41). These monomers act as haptens, small molecules that are not immunogenic alone but can elicit an immune response when bound to carrier proteins (6, 42).

Once bound, the hapten-carrier complex is recognized by antigen-presenting cells such as Langerhans cells and dermal dendritic cells, which migrate to lymph nodes and present the antigen to naïve T cells (43, 44). This process bridges the innate and adaptive immune systems, as the hapten induces local inflammation and cytokine release, creating conditions necessary for T-cell priming (43–46). Pattern recognition receptors, including Toll-like receptors (TLRs) and NOD-like receptors (NLRs), detect danger signals and further amplify the inflammatory environment (43, 44). Sensitization occurs during the immune system's first encounter with the hapten-protein complex, when dendritic cells present it to T-helper cells, stimulating B-cell activation and antibody production (47). This primes the immune system for a stronger response upon subsequent exposures. Repeated exposure can result in hypersensitivity, an exaggerated immune response that causes tissue damage (18, 47). Hypersensitivity reactions are classified into four types, with (meth)acrylates predominantly associated with type IV (delayed-type) T-cell-mediated reactions (6, 43, 48).

Type IV hypersensitivity has been linked to contact dermatitis, characterized by an eczematous rash with redness, pruritus, swelling, and dryness (2, 18, 49). In more severe cases, the rash may progress to blistering or skin fissures (41, 50). Although systemic reactions are rare, localized effects such as oral mucosal irritation and skin inflammation are more common (6, 41, 51). Symptoms typically appear within 24–48 h of exposure and can persist for weeks to years if exposure continues (6, 41, 51).

### Cytotoxic mechanisms

Residual (meth)acrylate monomers can produce various adverse biological effects, including irritation of the skin, eyes, and mucous membranes; allergic contact dermatitis; stomatitis; asthma; neurotoxicity; hepatotoxicity; and potential reproductive toxicity (2, 16, 20, 52).

A primary concern involves the capacity of these monomers to induce oxidative stress through elevated intracellular reactive oxygen species (ROS) levels, an effect documented for HEMA, Bis-GMA, TEGDMA, and UDMA (9, 30, 53, 54). While ROS play critical roles in normal cell signaling, excessive accumulation disrupts redox homeostasis, causing oxidative DNA damage, lipid peroxidation, and activation of pro-apoptotic pathways (9, 30, 53, 54). These effects raise concerns about potential genotoxic and mutagenic outcomes (30, 52, 55).

Beyond ROS elevation, monomer cytotoxicity proceeds through several interconnected mechanisms. Intracellular GSH depletion is an early event that promotes downstream oxidative damage, including mitochondrial membrane disruption, impaired ATP synthesis, and caspase-mediated apoptotic signaling (53, 56, 57). At the transcriptional level, HEMA alters expression of genes involved in oxidative defense, inflammatory signaling, and ECM organization, suggesting that sublethal exposure may impair cellular function even in the absence of overt cytotoxicity (58). Cells also activate adaptive responses to monomer-induced stress, particularly through Nrf2 pathway upregulation; however, cytotoxicity develops when exposure exceeds these protective mechanisms, with thresholds varying by monomer type, cell type, and individual susceptibility (54). Cytotoxicity generally increases in the order HEMA < TEGDMA < UDMA < Bis-GMA, reflecting differences in molecular weight, hydrophobicity, and membrane interaction capacity, and differs meaningfully across pulp cells, gingival fibroblasts, and oral epithelial cells (59–61).

These effects are concentration-dependent and greatest immediately after polymerization, when monomer release peaks (4, 20, 37). Translation to clinical settings remains difficult because salivary dilution, enzymatic degradation, and exposure duration influence tissue-level concentrations. In addition, *in vitro* findings are model-dependent, as Morisbak et al. demonstrated that cytotoxic responses to identical monomers varied substantially according to experimental conditions (62). Although substantial mechanistic evidence exists, the clinical relevance of (meth)acrylate cytotoxicity at concentrations encountered during routine dental treatment requires further investigation using standardized, physiologically relevant models.

### Cross-sensitization

Individuals who develop sensitivity to one (meth)acrylate monomer are at elevated risk of cross-reactivity with chemically related monomers (17, 41, 63–65). This phenomenon results from the structural similarity of (meth)acrylate haptens, which allows them to activate overlapping T-cell populations (43, 44). Strong cross-sensitization has been observed among HEMA, ethylene glycol dimethacrylate (EGDMA), and 2-hydroxypropyl methacrylate (HPMA), with HEMA recognized as a potent sensitizer capable of facilitating reactions to other (meth)acrylates not previously encountered (17, 63, 64, 66).

Despite chemical similarity, susceptibility varies between individuals; some dental personnel become highly reactive to multiple monomers while others remain unaffected despite similar exposure (17, 41, 63, 66). For sensitized individuals, strict avoidance of further exposure is critical to preventing exacerbation (41, 67, 68).

Beyond dental applications, (meth)acrylates are extensively used in cosmetic, medical, and industrial products due to their rapid polymerization, optical clarity, and mechanical strength (69). Common sources include artificial nail systems, UV-cured gels, surface coatings, orthopedic cements, and medical adhesives (6). As a result, sensitization to (meth)acrylates is not confined to dental personnel or patients but extends to a broad population of exposed individuals.

Occupational exposure is a major concern in non-dental environments. Cosmetologists frequently handle uncured acrylate gels during nail applications, exposing them to conditions that favor both dermal and respiratory sensitization (6). Industrial workers exposed to airborne MMA during manufacturing or coating processes are similarly at risk (2, 70–74). Reports of occupational asthma and contact dermatitis among these workers parallel those seen in dental professionals, reinforcing the notion that (meth)acrylate sensitization is a cross-industry issue (6, 17, 63).

These parallels have important clinical implications. Individuals who develop sensitivity through cosmetic or industrial exposure may subsequently react to dental adhesives or restorative materials containing similar monomers. This highlights the need for comprehensive allergy histories in dental settings, including questions about nail products, occupational exposures, and previous reactions to adhesives or medical devices (6). The widespread use of (meth)acrylates in consumer products also complicates source identification when allergic reactions occur, emphasizing the need for consistent labeling standards and increased awareness among clinicians and manufacturers (6, 20).

## Clinical manifestations

With ongoing advancements in dental materials and biomedical technology, the use of (meth)acrylate-based polymers has become increasingly prevalent (75). These compounds are essential in applications such as CAD-CAM, 3D printing, bonding agents, restorative composites, denture bases, and prosthetic devices (7, 75, 76). However, use of these materials has been associated with case reports of type IV hypersensitivity reactions involving various organ systems (41, 49, 51, 77–79).

### Dermal exposure

Dental professionals are at constant risk of (meth)acrylate exposure when handling uncured resins during restorative procedures (67, 68, 80). Case reports document dental students developing allergic contact dermatitis after contacting uncured resins, even while wearing gloves, with symptoms resolving after 1–3 weeks of topical corticosteroid treatment (41).

Neuropathic disorders resulting from dermal exposure to monomeric MMA have also been documented (2, 20, 71, 81, 82). MMA is a cutaneous irritant that penetrates the skin and causes numbness by impairing myelinated nerve function and reducing sensory conduction velocities in digital nerves (82). Individuals with long-term occupational exposure show increased prevalence of these neurological symptoms (2).

In clinical and laboratory settings, dermal exposure most commonly occurs during routine handling of uncured or partially cured resin-based materials (67, 68, 80). Although gloves are widely used, dental monomers can permeate common glove materials within minutes (see Clinical Safety section) (41, 67, 68, 83, 84). Exposure may also result from microtears, improper glove changes, contaminated surfaces, or direct skin contact during manipulation of resin-based materials (80). Emerging CAD-CAM and 3D printing workflows introduce additional pathways through handling of liquid or semi-cured resins (1, 7, 20). These practices can lead to repeated low-level exposure, contributing to sensitization in susceptible individuals (41, 67).

### Mucosal and systemic exposure

#### Oral mucosa

Oral mucosal exposure to resin-based dental materials can produce both local and systemic effects (20). Unpolymerized monomers such as TEGDMA, HEMA, and Bis-GMA can leach from resin-based materials, contact the oral mucosa directly, and exert cytotoxic effects (20, 85, 86). Due to the thin and highly vascularized nature of the oral mucosa, these monomers can be readily absorbed into systemic circulation, leading to adverse reactions such as oral lichenoid lesions, mucosal irritation, ulceration, endocrine disruption, and burning sensations (20, 40, 77).

Provisional crowns and bridges fabricated using PMMA-containing materials can trigger allergic reactions in patients (78, 87, 88). Symptoms may appear within 24 h and typically include burning sensation, swelling, redness, soreness, and erythematous ulcers at the contact site (77, 78, 87, 88). These symptoms generally resolved within days or weeks after the material was removed and replaced (77, 78, 87, 88). While these adverse mucosal reactions appear rare, with only isolated cases reported in the literature, they underscore the importance of recognizing (meth)acrylate sensitivity in clinical practice (77, 78, 87, 88).

Residual (meth)acrylate monomers may also exert cytotoxic effects on pulpal tissues, as studies have demonstrated that monomers such as Bis-GMA, UDMA, and TEGDMA can induce oxidative stress, glutathione depletion, and apoptosis in dental pulp cells (79). More recent investigations further suggest that resin monomers may contribute to mitochondrial dysfunction, inflammatory responses, and impaired odontoblastic activity within the pulp-dentin complex (56).

#### Ocular exposure

Eye and facial exposure to (meth)acrylate-based dental materials poses a significant occupational risk, particularly for dental professionals frequently exposed to monomer dust and vapors during resin handling, drilling, and curing (17, 70, 71, 73, 74). Among 126 dental personnel studied, 12%–15% of allergic reactions occurred on the face, neck, and eyelids, areas especially vulnerable to airborne or splash exposure (17). These reactions were most commonly linked to HEMA, MMA, and EGDMA (17). Dental technicians exhibited significantly higher risk compared to hygienists, with patch test positivity rates of 37% and 15.6%, respectively (17).

For both patients and practitioners, exposure to airborne (meth)acrylate particles can lead to sensitization and allergic contact dermatitis affecting the hands, eyelids, face, and conjunctival tissues, especially without appropriate protective measures (1). Allergenic particles may settle on the skin or be transferred from contaminated hands even without direct contact (17). Facial involvement, including ocular irritation and impaired vision, has been documented in cases of airborne dermatitis, emphasizing the importance of adequate ventilation and protective equipment (17).

#### Respiratory exposure

Inhalation remains the primary route of MMA exposure, with the lungs serving as the main site of metabolic clearance (70–74). Manipulation of dental composites during clinical procedures, particularly grinding and polishing, generates high concentrations of airborne nanoparticles in the breathing zone of both operator and patient (16, 89, 90). These particles ranged in median diameter from 38 to 70 nanometers, and electron microscopy confirmed they consisted of both inorganic filler and (meth)acrylate resin fragments (16, 89). Most particles generated during composite grinding are ultrafine (15–35 nm), with over 80% measuring less than 1 µm, well within the respirable fraction capable of reaching deep into the lungs (89–91). These particles likely consist of polymerized (meth)acrylate resin potentially containing residual monomers that may contribute to surface reactivity (16, 20, 89). Inhalation of these vapors and particles has been associated with coughing, respiratory irritation, asthma-like symptoms, pneumoconiosis, and emphysema (2, 20, 89).

A cross-sectional study involving 799 female dental assistants in Finland reported a significant association between daily (meth)acrylate exposure and increased incidence of adult-onset asthma, nasal symptoms, and work-related cough or phlegm (92). Similarly respiratory hypersensitivity reactions, including occupational asthma, rhinitis, and laryngitis, have been reported among dental personnel exposed to (meth)acrylate-containing materials (8, 93).

Clinical studies in dental students demonstrated moderate pulmonary function restriction following short-term MMA vapor exposure, with symptoms including coughing, mucus accumulation, and bronchospasm (70, 71). While these effects were reversible within days, ongoing exposure may lead to progressive respiratory impairment. Animal studies confirm significant pulmonary pathology following prolonged MMA inhalation (72, 74, 94, 95). The precise immunological mechanisms remain unclear, though current evidence suggests the reaction is not IgE-mediated (93, 96).

#### Emerging technologies

(Meth)acrylate-based materials play an increasingly prominent role in computer-aided design and computer-aided manufacturing (CAD-CAM) technologies in dentistry, particularly in the fabrication of prosthetic components, surgical guides, orthodontic aligners, and temporary restorations (7, 75, 97, 98). For instance, UDMA, a major component of many 3D-printed aligners, contributes to their flexibility and strength (76). CAD-CAM workflows may be subtractive (milling pre-polymerized blocks) or additive (3D printing with photopolymer resins). Both methods rely on (meth)acrylate-based materials, but additive manufacture depends heavily on the operator for achieving optimal final properties through proper printing parameters, post-curing protocols, and surface finishing (33, 75).

While these technologies offer precision and workflow advantages, they introduce numerous variables that influence polymerization quality and residual monomer release. Reported degree of conversion values for 3D-printed resins vary widely due to lack of standardization in evaluation methods. Some studies measure DC before washing; others after washing, which significantly affects results. Most evaluate only the surface, yet the internal core of the material often remains substantially less cured because UV light cannot adequately penetrate through different colors and opacities (33, 99). Post-processing protocols vary considerably in solvent type, washing duration, curing unit, and heat application, all of which influence final DC and monomer release (99). One study reported approximately 83% polymerization efficiency for 3D-printed dental resins, leaving nearly 17% of monomer content unreacted and capable of leaching into the oral environment (35). However, such values must be interpreted cautiously given these methodological inconsistencies. By contrast, CAD-CAM-milled materials, such as pre-polymerized PMMA blocks, are processed under controlled industrial conditions of high temperature and pressure (100). These conditions favor a higher degree of monomer conversion, contributing to longer polymer chains, lower residual monomer content, and reduced porosity (100). These differences contribute to why milled resins often demonstrate greater dimensional stability and lower residual monomer release compared to 3D-printed or chairside-cured materials (7, 33).

Given these methodological inconsistencies, the evidence on 3D-printed resin biocompatibility remains inconclusive. Some studies report cytotoxic effects before post-curing, while others find that optimized protocols render them comparable to traditional composites (7, 31). This highlights the urgent need for standardized printing and post-curing guidelines to ensure reproducibility and safety across clinical and laboratory settings.

## Clinical safety and protective measures

Daily exposure to (meth)acrylates, whether during placement, manipulation, or finishing, poses potential health risks if not managed properly (1, 41). Appropriate handling procedures have been shown to significantly reduce exposure levels (41, 80).

### Glove protection and limitations

The type of medical-grade gloves used in dental procedures plays a critical role in limiting exposure, although not all gloves provide equal protection (1, 20). Latex, nitrile, and vinyl gloves are all used in dentistry, but nitrile generally offers the greatest chemical resistance, while vinyl provides only basic protection (41, 101, 102). However, *in vitro* studies demonstrate that (meth)acrylates readily permeate all commonly used dental gloves, with breakthrough times of ≤2 min for MMA and approximately 6 min for HEMA (41, 83, 84, 103). *In vivo* patch testing further confirms that while nitrile gloves may prevent allergen penetration during brief exposures, protection is lost with exposures exceeding 30–60 min, with clinical reactions documented despite glove use (102). Permeation rates increase significantly when monomers are dissolved in solvents such as ethanol or acetone (41, 83, 103).

Glove degradation from sweating, prolonged wear, and monomer exposure reduces barrier integrity (41, 101, 102, 104). Latex and nitrile maintain integrity better than vinyl, with vinyl demonstrating failure rates of 12%–61% under simulated-use conditions compared to just 1%–3% for nitrile (105, 106). Dental gloves are designed for microbiological, not chemical, protection (102, 103). Gloves should be changed every 15–20 min during resin procedures, with double-gloving recommended for prolonged exposure (66, 102, 104).

Importantly, reduction of occupational exposure depends not only on glove selection, but also on proper glove use practices, including minimizing direct monomer contact, prompt replacement of contaminated gloves, and limiting prolonged exposure during resin procedures (41, 50, 66, 104, 107).

### Additional protective strategies

Additional protective strategies include disposable sleeves, barrier creams, moisturizers, and film-forming agents (107, 108). Dimethicone-based creams create hydrophobic layers that repel water-based irritants, while zinc oxide and other film-forming products provide physical shielding; however, protection decreases with moisture or solvent contact (50, 109). Barrier creams should be considered supplementary rather than replacements for gloves, as evidence regarding their protective efficacy remains variable and their effectiveness against permeating monomers is limited (50, 67, 107).

### Material selection and clinical protocols

To reduce intraoral risks, clinicians should be aware of the monomer composition of the materials they use, as lower-molecular-weight diluent monomers such as HEMA and TEGDMA generally demonstrate greater diffusion and elution potential (9, 59, 60). In practice, this involves reviewing product information sheets and material safety data sheets (MSDS) to identify the main resin monomers (110). However, MSDS information can be incomplete or misleading; analytical testing of a composite listed as Bis-GMA-free still detected small amounts of Bis-GMA, likely impurities below the 1% reporting threshold (111).

Unsafe practices such as placing uncured adhesive on gloved hands should be avoided (41). Adjusting curing protocols, including extended light-exposure times, may reduce detectable monomer release under certain conditions, however, reported outcomes remain variable depending on the material system, curing parameters, and study methodology (112). Broader precautions include the use of masks, protective eyewear, improved ventilation, airtight storage, automated dispensing systems, and routine hand washing after handling these substances (1, 50, 51).

### Respiratory protection

Proper respiratory protection may be beneficial when working with (meth)acrylate-based materials, particularly in laboratory or high-exposure settings (2). N95 respirators provide 8–12 times greater protection than surgical masks against submicron particles, though effectiveness depends on proper fit (113, 114). This is particularly important in digital dentistry workflows involving 3D-printed appliances, especially during post-processing procedures (16). Properly fitted respirators combined with effective ventilation and localized filtration are essential to minimize exposure (41).

### Hierarchy of controls

At the occupational level, (meth)acrylate sensitivity among dental personnel is increasingly recognized, with reported prevalence ranging from 10% to over 30% depending on population and exposure intensity (50, 115). PPE is the least effective control measure; higher-level strategies such as no-touch techniques, unit-dose dispensing, and engineering or administrative controls should be prioritized (66, 107). This aligns with the CDC's NIOSH Hierarchy of Controls, which ranks hazard reduction methods from most to least effective: elimination, substitution, engineering controls, administrative controls, and finally personal protective equipment (116, 117). In dentistry, this framework suggests that greater emphasis should be placed on upstream measures, including safer monomer formulations, closed or unit-dose delivery systems, and improved ventilation, to more effectively reduce occupational risks (116, 117).

## Conclusion

(Meth)acrylate-based materials remain indispensable in modern dentistry due to their mechanical properties, esthetic versatility, and compatibility with digital workflows. However, no current dental polymer achieves complete polymerization, and residual monomers can leach into the body through dermal, mucosal, ocular, and respiratory routes. These monomers act as haptens, capable of triggering type IV hypersensitivity reactions in both patients and dental professionals. Cross-sensitization among structurally related monomers, combined with widespread (meth)acrylate use in cosmetic and industrial products, further complicates exposure assessment and clinical management.

The rapid adoption of additive manufacturing technologies introduces additional concerns, as 3D-printed resins demonstrate significant variability in degree of conversion depending on post-processing protocols, and standardized guidelines remain lacking. Evidence on the biocompatibility of these materials is inconsistent, highlighting the need for methodological standardization in future research.

Clinically, protective measures such as gloves provide limited chemical barrier function against (meth)acrylate monomers. A hierarchy of controls approach, prioritizing elimination, substitution, and engineering controls over personal protective equipment, offers a more effective framework for reducing occupational risk. Moving forward, the development of low-allergenicity monomer formulations, standardized post-processing protocols for additive manufacture, and comprehensive allergy screening in dental settings will be essential to ensure patient and practitioner safety as (meth)acrylate use continues to expand. Future priorities should include regulatory consideration of clearer monomer content labeling, development of validated screening protocols for at-risk patients, and continued research into HEMA-free bonding systems.
