# Supplementary material for: Allergenic and cytotoxic potential of (meth)acrylate monomers in dental materials: a narrative review
Source: Front Dent Med. 2026 Jun 24;7:1846292. doi: 10.3389/fdmed.2026.1846292 (PMC13341657; doi:10.3389/fdmed.2026.1846292)
Supplement: Supplementary file 1 [file Supplementaryfile1.docx]

**Supplementary Table S1.** ISO 20795-1:2013 minimum requirements for denture base polymers.

| **Property** | **Heat-polymerized** | **Autopolymerized** | **Thermoplastic** | **Light-activated** | **Microwave-cured** |
| --- | --- | --- | --- | --- | --- |
| Flexural strength (min.) | ≥65 MPa | ≥60 MPa | ≥65 MPa | ≥65 MPa | ≥65 MPa |
| Flexural modulus (min.) | ≥2000 MPa | ≥1500 MPa | ≥2000 MPa | ≥2000 MPa | ≥2000 MPa |
| Residual MMA monomer (max.) | ≤2.2% | ≤4.5% | ≤2.2% | ≤2.2% | ≤2.2% |
| Water sorption (max.) | ≤32 µg/mm³ | ≤32 µg/mm³ | ≤32 µg/mm³ | ≤32 µg/mm³ | ≤32 µg/mm³ |
| Water solubility (max.) | ≤1.6 µg/mm³ | ≤8.0 µg/mm³ | ≤1.6 µg/mm³ | ≤1.6 µg/mm³ | ≤1.6 µg/mm³ |
| Color stability | Slight/no visible change | Slight/no visible change | Slight/no visible change | Slight/no visible change | Slight/no visible change |
